# The Potential of Metabolomics as a Tool for Identifying Biomarkers Associated with Obesity and Its Complications: A Scoping Review

**DOI:** 10.3390/ijms26010090

**Published:** 2024-12-26

**Authors:** Anna Katarzyna Skowronek, Marta Jaskulak, Katarzyna Zorena

**Affiliations:** Department of Immunobiology and Environment Microbiology, Medical University of Gdansk, 80-210 Gdansk, Poland; anna.skowronek@gumed.edu.pl (A.K.S.); marta.jaskulak@gumed.edu.pl (M.J.)

**Keywords:** metabolomic, obesity, type 2 diabetes, cardiovascular disease, hypertension, metabolic fatty liver disease, adipokines, amino acids, branched-chain amino acids

## Abstract

Obesity and its related diseases, such as type 2 diabetes (T2DM), cardiovascular disease (CVD), and metabolic fatty liver disease (MAFLD), require new diagnostic markers for earlier detection and intervention. The aim of this study is to demonstrate the potential of metabolomics as a tool for identifying biomarkers associated with obesity and its comorbidities in every age group. The presented systematic review makes an important contribution to the understanding of the potential of metabolomics in identifying biomarkers of obesity and its complications, especially considering the influence of branched-chain amino acids (BCAAs), amino acids (AAs) and adipokines on the development of T2DM, MAFLD, and CVD. The unique element of this study is the combination of research results from the last decade in different age groups and a wide demographic range. The review was based on the PubMed and Science Direct databases, and the inclusion criterion was English-language original studies conducted in humans between 2014 and 2024 and focusing on the influence of BCAAs, AAs or adipokines on the above-mentioned obesity complications. Based on the PRISMA protocol, a total of 21 papers were qualified for the review and then assigned to a specific disease entity. The collected data reveal that elevated levels of BCAAs and some AAs strongly correlate with insulin resistance, leading to T2DM, MAFLD, and CVD and often preceding conventional clinical markers. Valine and tyrosine emerge as potential markers of MAFLD progression, while BCAAs are primarily associated with insulin resistance in various demographic groups. Adipokines, although less studied, offer hope for elucidating the metabolic consequences of obesity. The review showed that in the case of CVDs, there is still a lack of studies in children and adolescents, who are increasingly affected by these diseases. Moreover, despite the knowledge that adipokines play an important role in the pathogenesis of obesity, there are no precise findings regarding the correlation between individual adipokines and T2DM, MAFLD, or CVD. In order to be able to introduce metabolites into the basic diagnostics of obesity-related diseases, it is necessary to develop panels of biochemical tests that will combine them with classical markers of selected diseases.

## 1. Introduction

Obesity is a pathological condition that involves excessive growth of adipose tissue and body mass in relation to height. As early as 1974, the World Health Organization (WHO) defined obesity as a disease of civilization and, in 2002, included it on the list of the greatest threats to humanity. According to the latest research, in 2022, over 1 billion people in the world suffered from obesity, of which over 300 million were people under the age of 18 [1]. The criterion for diagnosing obesity is defined by WHO guidelines. Obesity in adults is defined as a body mass index (BMI) > 30 kg/m^2^. In children, the BMI is additionally compared to centile charts appropriate for a given population, gender, and age. According to the current WHO guidelines from 2021, obesity in children under 5 years of age is determined based on a weight-for-height greater than three standard deviations above the WHO Child Growth Standards median, while in children aged between 5 and 19 years, obesity is greater than two standard deviations above the WHO Growth Reference median [2].

Obesity complications are significant and lead to a number of serious chronic diseases. In children, obesity often causes hormonal disorders such as hypercorticism and subclinical hypothyroidism, while in adults, it is primarily associated with cardiovascular diseases such as ischemic heart disease and ischemic stroke or even cancers, e.g., breast cancer and colon cancer. In addition, in both age groups, obesity causes insulin resistance (IR), leading to type 2 diabetes (T2DM), hypertension, metabolic-associated fatty liver disease (MAFLD), obstructive sleep apnea, and mental disorders, most often in the form of depression, and postural defects. Currently, methods of counteracting obesity and its effects include lifestyle modification, psychotherapy, bariatric surgery as a last resort, and pharmacotherapy, which is intended to slow down the process rather than stop it [2,3,4,5].

Due to the importance of obesity and its comorbidities in society, there is an increasing search for new parameters in the blood that could be early markers of obesity and the chronic diseases associated with it. The hope for this is seen in metabolomics, a new field of science that deals with the qualitative and quantitative analysis of low-molecular-weight metabolites that make up the body’s metabolome. Since metabolomics covers changes in the metabolome from the cellular level, through tissues, to entire systems and the organism, it seems that it can be a useful tool for monitoring early changes caused by obesity in cells and body fluids [6].

The latest literature reports that in organisms affected by the mentioned civilization disease, the greatest changes are observed in the concentrations and metabolism of amino acids (AAs), especially branched-chain amino acids (BCAAs). Additionally, important correlations are noted between the concentrations of individual metabolites and obesity-induced diseases. According to some scientific studies, high concentrations of BCAAs in fasting plasma may be a strong predictor of, among others, T2DM. Increasingly, attention is also paid to adipokines as possible markers of diseases associated with excess adipose tissue. These proteins have recently been studied in this respect, which is why the data on this subject are currently contradictory. However, it is worth expanding research on both adipokines and BCAAs and AAs and their potential relationship with obesity and comorbidities because these are parameters that can demonstrate high sensitivity and specificity in this area and can be new markers that will allow for the detection of disorders in the body earlier than conventional parameters [7,8].

The aim of the following paper is to systematically review the literature on the role of metabolomics in research on obesity and comorbidities in children, adolescents, and adults in the years 2014–2024. The research hypothesis assumes that specific metabolites correlate with the occurrence of obesity, and the observation of changes in their levels may play a significant role in the diagnosis and prevention of obesity-related diseases.

This systematic review introduces a new perspective by comprehensively examining the role of metabolomics in understanding obesity and its comorbidities across age groups, focusing on the last decade. A unique aspect of this study is the emphasis on identifying specific metabolites, such as BCAAs, individual AAs, and adipokines, as early biomarkers of obesity-related diseases. This research addresses critical gaps in the existing literature, including the limited understanding of metabolomic profiles in pediatric populations, particularly in relation to cardiovascular disease (CVD), and the lack of conclusive evidence linking individual adipokines to specific obesity-related complications, such as T2DM and MAFLD. By synthesizing recent findings, this review aims to provide a foundation for the development of standardized diagnostic approaches and to highlight the potential of metabolomics in the early detection and prevention of obesity-related disorders.

## 2. Methodology

The systematic review was developed based on a variety of publications that focus on the search for the role of metabolomics in obesity and obesity-related diseases in children, adolescents, and adults. The review included the results of studies published from 1 January 2014 to 30 June 2024.

The criteria for defining obesity were age-dependent but focused on the assessment of BMI and, in the case of people < 18 years of age, references of the BMI index to percentile charts appropriate for sex and age and in accordance with the WHO recommendation [2].

The search strategy included analyzing the literature contained in the PubMed and Science Direct databases. The search for relevant literature was possible thanks to previously developed keywords related to metabolomics, specifically BCAAs, AAs, and adipokines, and obesity, T2DM, MAFLD, and cardiovascular diseases in each age group. Keywords included: “metabolomics”, “obesity”, “children”, “adults”, “glycine”, “alanine”, “glutamine”, “valine”, “serine”, “lysine”, “phenylalanine”, “tyrosine”, “leucine”, “isoleucine”, “leptin”, “adiponectin”, “adipokines”, “cardiac disorder”, “liver”, “hypertension”, “blood pressure”, “diabetes”, and “T2DM”. Keywords were used individually and in collocations with “metabolomics obesity”.

Inclusion criteria: included only studies published in English, published from 1 January 2014 to 30 June 2024.

The selection of studies included only original studies, such as clinical trials, prospective studies, and retrospective studies. High-quality systematic reviews and meta-analyses were included in the introduction and general descriptions of the pathophysiology of obesity and chronic diseases to clarify metabolic pathways and provide a broader context for the problem.

**Figure 1:**
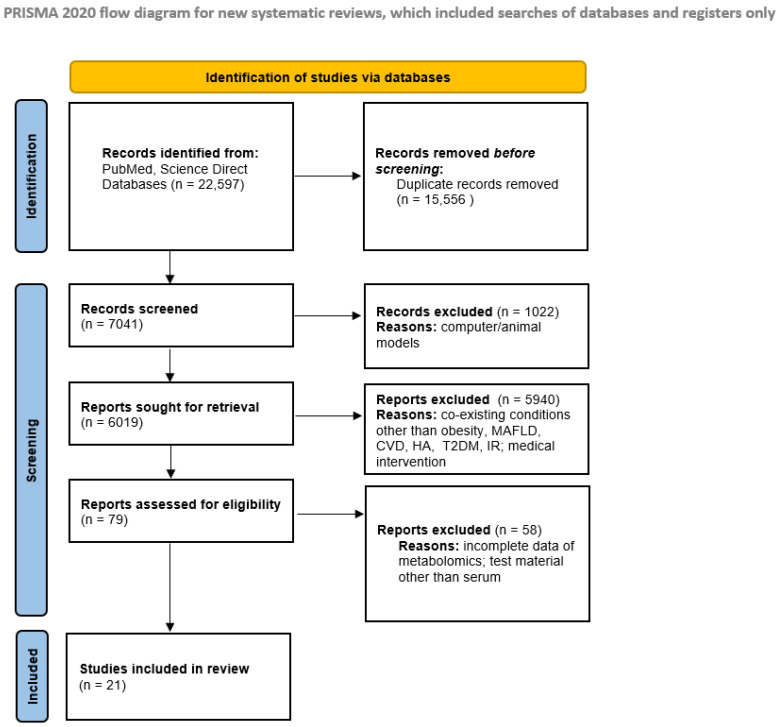
The PRISMA flowchart [9].

Studies were selected by one reviewer based on the inclusion, exclusion, and assessment criteria according to the PRISMA protocol. The PRISMA flowchart (Figure 1) illustrates the study selection process.

## 3. Adipose Tissue—Structure, Classification, Physiological Functions, and Changes in Obesity

Adipose tissue is a structure consisting of various cells, such as adipocytes, preadipocytes, endothelial cells, fibroblasts, and immune cells, including macrophages, dendritic cells, and T cells. All components of adipose tissue have an important secretory function, releasing metabolites, lipids, cytokines, and adipokines. In a physiological situation, adipose tissue participates in the homeostasis of the body by storing and releasing appropriate substances. However, in the case of excessive development of this tissue in the human body, homeostasis is disturbed through a change in the proportion between the synthesis of adipose tissue (lipogenesis) and the breakdown of adipose tissue (lipolysis) and hyperactivity of its secretory function [10,11,12].

Lipogenesis involves the synthesis of fatty acids, which are energy reserves. This process is stimulated by an increase in triglyceride levels, e.g., as a result of a diet rich in carbohydrates. In turn, lipogenesis is inhibited by the action of polyunsaturated fatty acids and a lack of food supply, which causes a decrease in plasma glucose levels and, thus, an increase in the level of free fatty acids. Additionally, lipogenesis is inhibited by leptin, a hormone produced by adipose tissue and responsible for the feeling of satiety [13].

Lipolysis is a process that involves a reduction in adipose tissue and the energy reserves (triglycerides) stored in it. In this way, energy is released from adipose tissue cells, and from a biochemical point of view, triglycerides undergo a process of hydrolysis, resulting in the formation of free fatty acids and glycerol. The former is bound by albumin and transported through the bloodstream to the liver, muscles, and other tissues for β-oxidation. This process is catabolic in nature and consists of the oxidation of free fatty acids to acetyl-coenzyme A. During this time, glycerol is transported to the liver, where it is oxidized or participates in glucogenesis, depending on the body’s needs [13].

Adipose tissue is classified into four types—brown, white, beige, and pink—but the most important are brown adipose tissue (BAT) and white adipose tissue (WAT). They can be distinguished macroscopically by color but also microscopically because the adipocytes in each type of tissue differ in physiology and function [11,14,15]. BAT is mainly responsible for the rapid conversion of stored energy into heat, which plays a key role in maintaining body temperature. For a long time, BAT was thought to occur only in infants, but studies from the early 21st century have shown the occurrence of this tissue in the supraclavicular and thoracic regions in adults as well [16,17].

However, WAT is the main adipose tissue that occurs in the body of children and adults. Due to its volume, wide distribution in the body, and versatility of roles, it is the focus of research on metabolomic changes in obesity. It performs a metabolic function, being the largest endocrine gland, an immunomodulatory function, a thermoinsulating function, and a protective function for internal organs. It has the ability to secrete numerous bioactive substances such as hormones, adipokines, enzymes, cytokines, growth factors, complement factors, and matrix proteins. It also contains numerous receptors that are key in processes such as food intake, energy expenditure, metabolism, body immunity, and blood pressure regulation. Through a complex network of endocrine, paracrine, and autocrine signals, this tissue influences the work of, among others, the hypothalamus, pancreas, liver, skeletal muscles, kidneys, endothelium, and the immune system [14,18,19].

Excessive WAT growth causes a number of changes in the functioning of adipocytes at the cellular level. The basis of these disorders is the impairment of mitochondrial function. As a result, the activity of metabolic pathways responsible for fatty acid oxidation, ketone metabolism, and the tricarboxylic acid cycle decreases. Such disorders result in improper differentiation of preadipocytes into adipocytes, adipocyte apoptosis, and fibrosis of adipose tissue. Defective adipose tissue stimulates the expression of genes encoding cytokines, chemokines, and adhesion molecules, which attract immunocompetent cells (primarily macrophages and lymphocytes) and initiate the synthesis of proinflammatory mediators. The resulting inflammation additionally impairs adipocyte metabolism. In the case of adipose tissue hypertrophy, changes in the expression of metabolites synthesized by this tissue are also observed. Among other things, a decrease in adiponectin concentration and an increase in leptin concentration are observed in obese people compared to people with a normal amount of adipose tissue [20,21].

In this article, the attention is focused especially on three groups of metabolites: adipokines, AAs, and BCAAs, which may potentially influence the metabolism of adipose tissue and their potential correlation with the main diseases caused by obesity, such as insulin resistance, T2DM, MAFLD, hypertension, and cardiovascular diseases.

### 3.1. Adipokine’s Role in Obesity

For a dozen or so years now, a possible relationship between adipokines, proteins produced and secreted by WAT, and obesity has been noted. These proteins are characterized by autocrine, paracrine, and endocrine effects on tissues and organs, due to which they can participate not only in the pathogenesis of obesity but also in the pathogenesis of chronic diseases associated with excessive growth of adipose tissue. Although several hundred adipokines have been discovered so far, in relation to obesity, attention is most often focused on the adipokines, resistin, visfatin, and leptin. This is because their effect on key metabolic, immunological, and inflammatory processes is the best known. Additionally, methods for their determination are available and relatively inexpensive compared to other adipokines.

Adiponectin is a polypeptide hormone that affects a number of metabolic processes. It is involved in the metabolism of glucose and fatty acids in the liver and muscles, thereby participating in the response of tissues to insulin. Its source is almost exclusively from adipose tissue, and in plasma, it takes three main forms: low-, medium-, and high-molecular-weight adiponectin. This adipokine affects vessels by inhibiting the adhesion of monocytes to endothelial cells; in addition, it reduces the transformation of macrophages into foam cells, inhibits the proliferation of smooth muscle cells, stimulates angiogenesis, and increases the synthesis of nitric oxide [22,23,24,25]. The changes in its concentrations are being looked for in terms of correlation with coronary heart disease, cardiovascular diseases, and insulin resistance in obese people [22,26].

Resistin is a cysteine-rich protein that is structurally similar to a cytokine. It is produced not only by adipocytes but also by immunocompetent cells and has been shown to be present in the bloodstream, bone marrow, lung, placenta, pancreatic islet cells, and adipose tissue cells. Insulin release appears to be triggered by inflammation, hyperglycemia, growth hormones, gonads, and IL-6. Resistin is also involved in insulin resistance, hence the name of the molecule, but it also appears to be a marker of obesity-induced inflammation, atherosclerosis, T2DM, and metabolic syndrome [27,28,29].

Visfatin is the only adipokine that directly participates in adipogenesis by engaging in the process of transformation of preadipocytes into adipocytes. This protein is synthesized mainly in adipocytes and macrophages of adipose tissue, but small amounts are also produced in hepatocytes and neutrophils [30]. Additional functions of visfatin include stimulating pre-β lymphocyte colonies, increasing the synthesis and storage of triglycerols in adipose tissue, stimulating the synthesis of nitric oxide, which causes blood vessel dilation, and influencing the proliferation of endothelial cells. In the case of this adipokine, the most sought-after correlations are with the occurrence of insulin resistance in the course of obesity [31,32,33].

Leptin, the satiety hormone, is synthesized in mature WAT adipocytes, from where it is released into the bloodstream. The intensity of its synthesis and secretion depends on the volume of WAT and the size of adipocytes [26,34,35,36]. When released into the bloodstream, it is transported to the brain, where it binds to receptors in the hypothalamus. In this way, it inhibits genes encoding neuropeptide Y (NPY) and induces genes encoding proomyomelanocortin (POMC) and coticoliberin. The effect of this action is to inhibit appetite and generate a feeling of satiety, consequently reducing food consumption and limiting the growth of adipose tissue, and in the long term, even reducing the mass of adipose tissue [35]. Leptin is also considered a cytokine because it is involved in the body’s immune response by activating macrophages and natural killer cells (NK cells). Additionally, its functions include the regulation of proliferation, phagocytosis, chemotaxis, and the release of oxygen radicals by neutrophils [34]. This adipokine also participates in thermogenesis by regulating mitochondrial proteins specific to brown adipose tissue. Through the pleiotropic effects of leptin, it is also believed to be involved in the control of blood pressure, increased tissue sensitivity to insulin, and increased glucose uptake and oxidation in skeletal muscle [37,38,39].

### 3.2. AA’s and BCAA’s Role in Obesity

The group of amino acids contains various organic compounds, but the common feature is the molecular structure, distinguished by the simultaneous content of a basic amino group and an acidic group. The division of amino acids can be based on origin, chemical structure, or their role in the body. In this article, attention is focused on selected protein amino acids, which, according to the available literature, seem to be most closely related to the metabolism of adipose tissue. The roles of glycine, alanine, glutamine, valine, serine, lysine, phenylalanine, tyrosine, leucine, and isoleucine are investigated. From the broad group of AAs, we can also distinguish the group of BCAAs, which include leucine, isoleucine, and valine [40,41,42,43,44].

It has long been known that amino acids play key roles in the human body, such as participating in the synthesis of proteins, hormones, purine bases, and neurotransmitters, but they also participate in the regulation of biological processes, including their influence on lipogenesis. To understand this correlation well, it is worth taking a closer look at intracellular metabolic pathways, especially the Krebs cycle. This metabolic pathway connects all the basic amino acids directly and indirectly, especially the amino acids observed in this research. The Krebs cycle, or the tricarboxylic acid cycle (TCA) or citric acid cycle, is the final and common pathway for the oxidation of energy substrates, which provides energy in the form of ATP and metabolites for subsequent biosynthetic pathways. In relation to adipose tissue, the TCA cycle influences lipogenesis and lipolysis depending on the metabolic and nutritional needs of the body. The basic substrate of the cycle is acetyl-CoA, which can be formed as a result of the metabolism of leucine, tyrosine, lysine, phenylalanine, or isoleucine. In the course of the TCA cycle, acetyl-CoA is condensed with oxaloacetate, resulting in the formation of citrate, which participates in the subsequent stages of the TCA cycle. When the Krebs cycle is saturated, the increase in ATP concentration in the cell causes the cycle to self-limit and the associated transformations to be inhibited. At this point, citrate formed from the transformation of acetyl-CoA is redirected to the lipogenesis and fatty acid synthesis pathway [40,41,42].

Glutamine is also associated with the Krebs cycle, which, as a result of reductive metabolism, provides carbon for the synthesis of fatty acids when there are no other metabolites of the TCA cycle. While citrate is redirected to the lipogenesis pathway, glutamine, through its metabolite α-ketoglutarate, guards the balance of the TCA cycle and participates in lipogenesis [43,44].

In the case of glycine, serine, and alanine, they are often referred to as glucogenic amino acids because the direct or indirect metabolite of the catabolism of these AAs is pyruvate, which is part of the gluconeogenesis pathway. In gluconeogenesis, non-sugar precursors are converted into glucose. Glycerol is also used in this metabolic process, which, together with fatty acids, is a product of lipolysis. Additionally, some of the above-mentioned AAs can be converted into each other through reversible reactions during metabolism. In this way, serine can be metabolized into pyruvate in a two-track reaction by dehydration or converted into glycine by serine hydroxymethyltransferase. At the same time, glycine can supplement serine deficiencies by undergoing a reaction in the opposite direction to the one described above or be decomposed into CO_2_ and NH_3_ in liver mitochondria. In this way, the metabolism of glycine and serine is closely linked to each other and to the metabolism of adipose tissue [45,46,47].

Many studies have shown that BCAAs also regulate food intake, energy expenditure, the functions of hormones such as insulin, leptin, and glucagon, and tissue sensitivity to insulin [48]. The basic mechanisms of action of these amino acids are still not fully understood, but it is known that BCAA catabolism via the intermediate metabolite leucine promotes lipogenesis of subcutaneous WAT in obesity. The BCAA catabolic pathway is complex and only partially shared by all three BCAAs. The first two steps of this pathway are identical for isoleucine, leucine, and valine. BCAA aminotransferase catalyzes transamination, resulting in the formation of branched-chain keto acids (BCKAs). The next step is catalyzed by the branched-chain ketoacid dehydrogenase complex. The kinase of the complex phosphorylates one of its subunits to inhibit its activity. In the next step, each BCAA goes through its own individual catabolic pathway with other enzymes and processes. Both BCAAs and their catabolites perform important metabolic functions [49]. In this way, one of the products of valine catabolism (3-hydroxyisobutyrate) regulates the uptake of fatty acids by blood vessels and influences the sensitivity of muscle tissue to insulin [48]. As mentioned above, one of the products of leucine metabolism is acetyl-CoA, which not only enters the Krebs cycle but can also activate mTORC1 signaling [40,50,51]. By activating the mTORC1 complex, acetyl-CoA can affect the growth and metabolism of other cells, including taking part in adipogenesis by controlling lipid metabolism and adipocyte synthesis. Dysregulation of mTORC1 is common in various diseases, e.g., cancer or diabetes. Importantly, it is the mTORC1 complex that, right after the TCA cycle, connects the BCAA and AA metabolic pathways in adipose tissue.

BCAAs, particularly leucine, isoleucine, and valine, have emerged as significant predictors of insulin resistance and T2DM. Elevated BCAA levels have been observed to precede conventional clinical markers such as fasting glucose and HbA1c in at-risk individuals, and environmental factors such as exposure to endocrine-disrupting chemicals (EDCs) may exacerbate these disruptions in metabolic pathways. By translating these findings into clinical practice, BCAA levels could be integrated into early screening protocols to identify individuals at higher risk for metabolic dysregulation. Additionally, dietary interventions aimed at modulating BCAA intake or enhancing their metabolic clearance may offer new therapeutic pathways. For instance, increasing physical activity, which enhances BCAA catabolism in muscle, could complement pharmacologic approaches. The literature highlights significant gender differences in metabolite levels that impact obesity-related pathologies. In males, higher baseline BCAA levels have been linked to greater muscle mass and androgen-driven metabolic activity. Testosterone appears to enhance BCAA utilization, potentially contributing to increased insulin resistance when the BCAA metabolism becomes dysregulated. In females, the protective effects of estrogen on glucose metabolism may partially explain lower BCAA levels and reduced susceptibility to early insulin resistance [46,47,48,49,50].

Leptin and adiponectin demonstrate distinct clinical utility in obesity-related pathologies. Elevated leptin levels correlate with appetite dysregulation and could be targeted by therapies designed to enhance leptin sensitivity. Conversely, reduced adiponectin levels, commonly observed in individuals with obesity and cardiovascular diseases, suggest a role for adiponectin as a biomarker for cardiometabolic risk [52]. Adipokines, such as leptin and adiponectin, also exhibit gender-dependent behaviors. Leptin levels are typically higher in females, reflecting greater fat mass and its regulatory influence on reproductive hormones. However, resistin and visfatin, implicated in insulin resistance and inflammation, display varying correlations with metabolic markers based on gender. These observations underscore the need for gender-specific diagnostic thresholds and interventions. Environmental pollutants, including heavy metals, may also influence adiponectin concentrations, compounding their effects on metabolic health. Efforts to develop adiponectin-mimetic drugs or interventions to increase endogenous adiponectin secretion represent promising avenues for treatment [53].

Based on those findings, a composite diagnostic panel incorporating BCAAs (e.g., valine, isoleucine), aromatic amino acids (e.g., phenylalanine, tyrosine), and classical markers (e.g., fasting glucose, ALT) could significantly enhance diagnostic accuracy for MAFLD and other obesity-related complications. For example, higher valine levels have been strongly associated with progressive liver fibrosis, suggesting its inclusion in MAFLD-specific panels. Such panels could guide early therapeutic decisions, reducing the risk of irreversible complications like liver cirrhosis.

During childhood and adolescence, hormonal and metabolic shifts significantly influence metabolite levels. Puberty, characterized by increased sex hormone production, alters BCAA metabolism and insulin sensitivity. Additionally, environmental exposures during this critical developmental period, such as air pollution, may further disrupt metabolic processes and exacerbate obesity-related complications. Studies indicate that adolescent males exhibit a stronger correlation between elevated BCAA levels and insulin resistance compared to females, likely due to testosterone’s metabolic effects. In younger children, progressive obesity is associated with declines in glycine and serine levels. These changes reflect disruptions in gluconeogenesis and fatty acid metabolism, suggesting that interventions targeting these pathways could be particularly effective in pre-pubertal populations. Early identification of such metabolic shifts through age-stratified metabolomic panels could inform timely preventive measures.

## 4. The Role of Metabolomics in T2DM, MAFLD, HA, and CVD

### 4.1. T2DM and Insulin Resistance in Obesity—The Role of Metabolomics

In 2014, Newbern D. et al. conducted a study on a group of 82 obese adolescents (12–18 years of age) examining the associations between metabolites and glucose metabolism disorders. In bivariate analyses, adiponectin correlated negatively with insulin and Homeostasis Model Assessment of Insulin Resistance (HOMA-IR) in boys, while in girls, it did not show any correlation with any of the variables. Additionally, in the same analyses, leptin correlated with BMI in both girls and boys, but at the same time, it did not show any correlation with HOMA-IR or adiponectin. However, in the multivariate principal component analysis (PCA), a positive association of HOMA-IR with BCAAs (*p* = 0.0015) and glucogenic amino acids—proline and alanine—(*p* = 0.0043) was noted in the entire cohort. Regression models were also analyzed, taking into account gender, which showed that HOMA-IR in boys correlated positively with BCAAs, but such correlation did not appear in girls. Additionally, regardless of gender, age, and BMI, a negative correlation between BCAAs and adiponectin was demonstrated; however, after stratification by gender, this correlation remained statistically significant only in the male group (*p* = 0.008). Based on the obtained results, the authors of the study drew the following conclusions: BCAA levels and their metabolic products are higher in obese adolescents in the male group than in the female group; BCAA metabolites correlate with insulin resistance in this age group; and the relationship between HOMA-IR, adiponectin, and AA metabolism shows differences depending on gender. Such results suggest that gender plays a significant role in the pathogenesis of IR during puberty when major hormonal changes occur in the body [54]. A summary of the role of metabolites and complications in the course of obesity based on the available literature is presented in Table 1.

A year later, a study by Lee et al. was published on a population of Korean boys. The study was consistent with the observations of Newbern D. et al. The researchers included a 2-year follow-up of a group of 109 boys aged 9–11 years, from whom parameters, including serum, were collected at the beginning and at the end of the study. Already at the beginning of the study, higher concentrations of BCAA (isoleucine, *p* = 0.0002; leucine, *p* = 0.0002; and valine, *p* < 0.0001) and AA (phenylalanine, *p* = 0.0164; and tyrosine, *p* = 0.0002) were found in obese children than in nonobese children. After 2 years, these differences remained, except for phenylalanine, for which the correlation was no longer statistically significant (*p* = 0.1875). At the same time, it was noted that the level of glycine and serine, amino acids considered to be glucogenic, decreased with the progression of obesity. Next, an attempt was made to determine which metabolites correlated most strongly with HOMA-IR. A positive relationship with HOMA-IR was initially demonstrated by isoleucine and valine, while leucine, phenylalanine, and tyrosine showed a positive correlation with metabolic disorders but lost it after taking into account obesity parameters. Logistic regression analysis was also performed to check whether there is a correlation and what is the nature of the correlation between metabolite levels at the beginning of the study and the risk of insulin resistance in the future. The analysis showed that only BCAAs have a significant risk correlation with HOMA-IR, taking into account age and obesity parameters. Therefore, it was concluded that high BCAA concentration increases the risk of insulin resistance thrice during the 2-year observation period. Additionally, they observed that obesity in children under 10 years of age may lead to metabolic profile disorders and then to future pathogenesis of metabolic diseases. Lee et al. also stated in their study that the above results seem to indicate that the increase in BCAA is caused not only by obesity but also by the disruption of metabolic pathways in the course of excessive development of adipose tissue [55].

In the same year, Tillin et al. published their prospective research conducted over a 19-year period on a large group (2286 individuals) of adult men from Europe and South Asia. The researchers were looking for the correlations between BCAAs (isoleucine, leucine, and valine), AAs (tyrosine, alanine, glutamine, glycine, and histidine), obesity, and glucose metabolism disorders in different ethnic groups. First, they noted differences between each ethnic group—men from South Asia were more likely to have insulin resistance and arterial hypertension (HA) and also to have central obesity, with a lower BMI than Europeans. They also showed less physical activity and consumed less meat, fish, and dairy products. In the baseline metabolite study, higher levels of isoleucine, phenylalanine, tyrosine, and alanine were observed in men from South Asia than in men from Europe. Regardless, during the analysis of the correlation of AAs with markers of glucose metabolism disorders, positive correlations were observed with these parameters for isoleucine, leucine, valine, phenylalanine, tyrosine, and alanine in each ethnic group. Additionally, in both ethnic groups, glycine and glutamine were found to be negatively correlated with markers of glycemia and insulin resistance. Interestingly, the described correlations were insignificantly weakened in the group of men from South Asia. The authors suggested that such research results may lead other scientists to the path of observation of branched and aromatic AAs (especially tyrosine) in the South Asian population because, in this cohort, they seem to play a major role in the pathogenesis of insulin resistance and T2DM [56].

In line with the above results, in 2016, Chen et al. published their results of the study on a population of 429 Chinese adults. They found that leucine, isoleucine, valine, phenylalanine, and tyrosine could be biomarkers of T2DM. The team conducted two nested studies, which showed significantly increased levels of the above BCAAs and AAs in the group of diabetics than in the group of healthy individuals. These results were statistically significant and characterized by fold changes higher than two. Of these five metabolites, special attention was paid to the strong correlation between valine and the risk of developing T2DM (*p* < 0.001). Additionally, the correlation between AAs and conventional metabolic markers was examined, which showed a strong positive correlation of AAs with markers, especially with HOMA-IR. In the longitudinal study, the researchers showed that the baseline levels of the studied AAs were significantly increased in people diagnosed with T2DM 10 years later. In addition, this study noted an earlier increase in blood AAs than conventional clinical markers in response to T2DM. However, in a second analysis, the same AAs did not show as strong a correlation as clinical metabolic markers [57].

However, in the same year as Chen et al., Honda et al. published an article, the results of which are partially contradictory to the above studies and shed new light on the effect of BCAA on insulin resistance and T2DM in obese people. Honda et al. conducted a study on a group of 78 adult Japanese citizens, during which, both in univariate and multivariate analysis, they noted a positive effect of BCAA and tyrosine on tissue sensitivity to insulin in non-diabetic people. Interestingly, in multivariate regression models, the researchers noted a positive correlation between BCAA and HOMA-beta and a negative correlation of BCAA with HOMA-IR in men. At the same time, in the same analysis in women, they showed a negative correlation of tyrosine with HOMA-beta. In their work, BCAA in women did not show a correlation with HOMA-IR. Based on the results, according to which BCAA concentration showed a beneficial and adverse effect on glucose and lipid metabolism, the researchers put forward a hypothesis that this may be due to the mutual influence of these metabolites. Additionally, they found that after introducing prediabetes into the analysis, the correlation between BCAA and HOMA-IR weakened (regression coefficient/t/p/95% CI, −7712.88/−2.53/0.0133/−13,777.98 to −1647.80). According to them, BCAA may modulate tissue sensitivity to insulin by increasing fasting glucose and insulin levels, which may contribute to the conflicting results of studies on the correlation between BCAA and insulin resistance. For this reason, they suggest that univariate analyses regarding the above correlations should be interpreted with caution. They also noted that when analyzing metabolic markers, the gender of the examined person should be taken into account because sex hormones affect the development of insulin resistance and obesity. Moreover, when analyzing the group of men and women separately, it was noticed that the concentration of BCAA and tyrosine in men is initially higher than in women, which may also be related to the greater muscle mass developed in men. Multivariate analysis showed that BCAA positively correlates with muscle mass. It is likely that large muscle mass also facilitates glucose uptake and oxidation of free fatty acids. Thus, it was emphasized that it is important to determine which BCAAs affect the increase in muscle mass and reduce insulin resistance. In their study, the researchers also took into account parameters such as AST and ALT, which may indicate liver function. In their analysis, BCAA and tyrosine significantly correlated with AST but not with ALT. Therefore, they suggested that protein catabolism and skeletal muscle degradation may affect AST levels but not liver function. They also noted that it is worth conducting a control study on the optimal supply of BCAA with food to have a beneficial effect on insulin metabolism [58].

In 2016, Haufe et al. also tested the hypothesis that increased BCAA and aromatic AA concentrations are markers of insulin resistance and excessive fat deposition, among other things, in the liver. In their study, they noted that women have lower BCAA levels than men, which could confirm the influence of gender on BCAA metabolism, as was already noted in the above research studies. Additionally, in the study by Haufe et al., a correlation was noted between tissue sensitivity to insulin and BCAA and AA concentrations (*p* < 0.05); however, after taking into account age, gender, and BMI, the correlation of tissue sensitivity to insulin was lost for aromatic AAs. Additionally, in the univariate test, a correlation was noted between liver insulin resistance and BCAA concentrations (*p* < 0.01); after stratification by gender, age, and BMI, statistical significance in this correlation was maintained for isoleucine and valine [59].

In 2017, Goffredo et al. conducted a study on a group of 78 children aged 10–16 years of different ethnic origins living in the USA. For statistical analyses, they used the Spearman test with the Spearman rank correlation coefficient (r) and random forest (RF) analysis. They noted a negative correlation between tissue insulin sensitivity and isoleucine levels (r = −0.318; *p* = 0.005) and valine (r = −0.369; *p* = 0.001) for the entire study population. This is consistent with previous studies that BCAAs had a negative correlation with tissue insulin sensitivity and a positive correlation with HOMA-IR. Goffredo et al. also conducted a hyperinsulinemic-euglycemic clamp test, in which they showed a relationship between BCAA and the percentage inhibition of glucose production in the liver. They thus noted a negative correlation between BCAA and peripheral and hepatic insulin sensitivity, which had already been noted by Haufe et al. [60].

The association of BCAA with insulin resistance and the development of T2DM was also noted by Flores-Guerero et al. in their study on a large population of adult Dutch citizens (6244 people). The researchers showed in both crude and univariate and multivariate analyses that the BCAAs studied by them (leucine, isoleucine, and valine) were positively associated with HOMA-IR. At the same time, no association was found between BCAA and the function of pancreatic β-cells, which was reflected by the calculated HOMA-β index. Flores-Guerero et al. noted that patients with high levels of BCAA in serum presented a significantly higher risk of T2DM, and this correlation remained significant after adjustment for risk factors such as age, gender, BMI, family history of T2DM, hypertension, alcohol consumption, HOMA-IR, and HOMA-β. Additionally, an improvement in the predictive ability of T2DM was observed in the traditional prediction model after adding BCAA to it [61].

In 2022, a prospective study by Perng et al. on the role of metabolomics in childhood obesity was published. Their work was based on collecting a cohort of 179 Mexican children aged 8–14 years and searching for biomarkers of metabolic disorders among BCAAs over a 5-year observation period. Additionally, the innovativeness of the work is influenced by the use of the Least Absolute Shrinkage and Selection Operator (LASSO) regression for statistical analyses, which allows for the validation of results based on hypotheses and enables multivariate analysis. The researchers found the strongest correlation between isoleucine, tyrosine, and phenylalanine and insulin resistance. Additionally, they noticed a difference in the relationship between metabolites and insulin metabolism among pre-pubertal and pubertal girls. In the former, they did not observe any significant correlations, while in adolescent girls, they noted a positive association between BCAAs, especially leucine and phenylalanine, and C-peptide, which is a by-product of the conversion of proinsulin to insulin. In their work, Perng et al. also observed a positive correlation of leucine, isoleucine, tyrosine, and alanine with leptin in adolescent girls. The network of connections between BCAAs, C-peptide, and leptin seems obvious when one considers that insulin and leptin regulate food intake and participate in glucose homeostasis. In this study, the determinant of metabolic disorders was insulin resistance dependent on C-peptide; however, in the male group, the results remained consistent with the results of Honda et al.—the BCAA pattern correlated with reduced insulin resistance in this group. The authors noted the inverse relationship between isoleucine and the development of insulin resistance, which may confirm earlier assumptions that this BCAA affects glucose uptake by skeletal muscles and participates in glucose oxidation in the whole body. It was suggested that a higher level of isoleucine in serum could reduce the level of circulating insulin and thus be a prophylactic against insulin resistance. Interestingly, the LASSO method did not select BCAA as the strongest predictor of metabolic disorders among the various metabolites analyzed in this work. This may be due to the fact that when taking into account all metabolites, not only BCAAs, when estimating beta for the correlation between each compound and the change in the levels of conventional biomarkers, the beta values for BCAA represented marginal associations. Thus, it can be concluded that BCAAs positively affect insulin synthesis and activation. At the same time, the authors draw attention to the need for further studies on metabolomics and metabolic disorders in children and adolescents in relation to the period of puberty [62].

### 4.2. MAFLD in Obesity—The Role of Metabolomics

Obese individuals are at risk for fatty liver disease, which is characterized by liver inflammation and hepatocyte necrosis with varying degrees of fibrosis. The pathophysiology of this disease is complex and involves various mechanisms and multiple genetic, epigenetic, and environmental factors [68]. Disturbed metabolism of adipose tissue in obesity causes the release of excessive amounts of free fatty acids, which are transported to the liver and increase lipogenesis. Additionally, in obesity, tissues are less sensitive to insulin, which begins to be produced in excess. Through the portal vein, the liver is intensified to high concentrations of insulin, which also contributes to lipogenesis [59].

In 2016, Haufe et al. searched for the association between AA and BCAA and MAFLD and insulin resistance in a cohort of 111 obese adults. They showed that increased BCAA and aromatic AA (except phenylalanine) concentrations significantly correlated with increased intrahepatic lipid accumulation, and this correlation was maintained after adjustment for age, sex, and BMI. The authors of the study see great potential in studying the effect of BCAA and aromatic AA on ectopic fat storage in the liver and hepatic insulin resistance [59].

In the same year, Jin et al. published a paper on a pilot study of a small group of obese Latino adolescents, attempting to assess the impaired metabolic pathways in individuals with MAFLD based on the assessment of their metabolic profile in serum. In their study, they noted that the most dysregulated metabolism seemed to concern tyrosine, which showed a strong positive association with MAFLD even after taking into account age, gender, BMI, insulin, and HOMA-IR. Additionally, dysregulation was noted in the metabolism of BCAA, glycine, serine, and alanine, but these results were not as significant as for tyrosine. Additionally, it was not possible to separate the relationship between these metabolites and insulin resistance, in which higher levels of BCAA are also noted [63].

A similar study objective was also chosen by Goffredo et al., who conducted a study on a group of 78 children aged 10–16 years of different ethnic origins living in the USA. In the analysis conducted by random forest (RF), they showed that obese people with MAFLD have higher levels of valine, leucine, and isoleucine than obese people without MAFLD, which is consistent with the conclusions of Haufe et al. After taking into account factors that may confound the analyses (sex, age, ethnic origin, BMI, and insulin sensitivity), the correlations for valine (*p* = 0.02) and isoleucine (*p* = 0.03) remained statistically significant. Additionally, they assessed the correlation between metabolite levels and changes in liver fat content during the 3-year follow-up. They noted that higher baseline levels of valine were noted in children whose liver fat content increased during the follow-up period than in those whose liver fat level remained stable or decreased. Thus, it seems that valine may be a predictive marker of MAFLD. In their study, they did not include an assessment of tyrosine levels, but the observation of higher BCAA levels in people with MAFLD is consistent with the results of the two above-mentioned studies [60].

In 2018, the work of Romero-Ibarguengoitia et al., who conducted research on 137 adults in Mexico, was published. They divided the subjects into three groups: the group with normal BMI, the group with obesity but without MAFLD, and the group with obesity and MAFLD. In the first analyses, the ANOVA test and the Fisher post hoc test, they noted higher concentrations of alanine, leucine, phenylalanine, tyrosine, and valine in obese people with MAFLD compared to the group of obese people without MAFLD. Additionally, the researchers conducted analyses on structural equation models assessing the causal relationships between phenotype, metabolomics, inflammatory markers, and family history of obesity. In one of the models, they noted an interesting cause–effect sequence based on the model of obesity resulting from a disturbed balance between energy supply and expenditure. In this model, increased amino acids predicted increased alpha-keto acids with subsequent increased acylcarnitines, which led to, among other things, MAFLD and insulin resistance [65].

In the same year, the work of Gaggini et al. was also published, which was based on a study conducted on a population of 64 Italian adults who were divided into a group of healthy people, obese people with MAFLD, and lean people with MAFLD. The study showed that BCAA concentration positively correlates with obesity and MAFLD, and the data are statistically significant (*p* < 0.05). In obese people with MAFLD, changes in the concentrations of amino acids that play a key role in glutathione synthesis were also observed—glutamate levels were increased, while serine and glycine levels were decreased. Based on these data, a new GSG index (glutamate to serine to glycine ratio) was proposed, which may reflect the stage of MAFLD, especially in the context of liver fibrosis [64].

Lischka et al. went a step further, first showing in a cohort of 68 children that BCAAs (*p* < 0.01) were significantly correlated with liver fat content even after adjusting for sex, age, and pubertal stage and then confirming this correlation with ANOVA analysis. However, because of the limited analytical value of BCAAs when considered in isolation, the researchers created a linear regression model that could predict MAFLD. After adjusting the model for age, sex, and pubertal stage, it was defined as follows: Predicted MRI-PDFF = BCAA (μmol/L) × 0.03 + ALT (U/L) × 0.144 + GGT (U/L) × (−0.208) + ferritin (μg/L) × 0.041 + insulin (μU/mL) × 0.313–14.04. Then, the model was subjected to c-statistical analyses (AUROC) and c-statistics. Thus, it was shown that the BCAA-based model presented 91.7% sensitivity for mild fatty liver disease, and the specificity remained at 35%. The researchers indicated that BCAAs may be an important link between obesity, MAFLD, and other metabolic disorders, so further studies based on these metabolites should be conducted [66].

Chae et al. conducted research on a population of 165 Korean children who were divided into four groups: obese individuals with MAFLD, obese individuals without MAFLD, lean individuals with MAFLD, and healthy individuals without obesity and MAFLD. Such a division allowed for the identification of the metabolic profile of individuals with MAFLD resulting from obesity. During the study, it was observed that the level of BCAA, lysine, and tyrosine was significantly higher in obese individuals with MAFLD than in obese individuals without MAFLD. To exclude the participation of insulin resistance in the modeling of metabolite levels and to show a specific correlation with MAFLD, metabolic features were compared with clinical features. Thus, it was shown that some metabolites had a weak association with insulin resistance. Due to the lack of difference in the HOMA-IR level in the healthy group and the lean group of MAFLD subjects and the higher HOMA-IR level in the obese group with MAFLD than in the obese group without MAFLD, it was assumed that the studied metabolites are specific for fatty liver disease. Additionally, information was sought about which metabolic pathways are altered in obese MAFLD subjects. For this purpose, enrichment analysis of the metabolite set based on SMPDB was performed, which showed pathways altered in the group of obese patients with MAFLD compared to the group of obese subjects without MAFLD: valine, leucine, isoleucine metabolism, alanine metabolism, and glutathione metabolism (enrichment factor > 4, raw *p* < 0.05). Mapping of metabolic features typical for MAFLD was also performed based on MetaMapp, during which upregulation of tyrosine and glutamic acid expression and downregulation of glycine were observed in obese subjects with MAFLD. The researchers also noted an upregulation of plasma BCAA levels in obese individuals with MAFLD. These results are consistent with the work cited above in both adult and pediatric populations. Furthermore, based on the logistic regression model coefficients and the results of the significance of variables in other models, the researchers identified selected metabolites as significant in predicting the development of MAFLD in the course of obesity: valine, tyrosine, glutamic acid, and glycine [67].

Garibay-Nieto et al. focused their study on the possibility of distinguishing two stages of MAFLD (steatosis and fibrosis) based on metabolomics. They conducted a study on a cohort of 79 Mexican children aged 8–16 years, in whom the levels of alanine, glycine, valine, leucine, phenylalanine, and tyrosine were measured, and the correlation of changes in the levels of these metabolites with the occurrence of one of the two stages of MAFLD was sought. They performed a series of statistical analyses, such as VIP and ROC analysis, but did not reveal statistically significant differences between the studied groups. In the VIP analysis between measurements made in patients without MAFLD and patients with MAFLD, it was noted that glycine may be a variable distinguishing these groups (VIP > 1.5). In the same analysis between measurements from individuals with different MAFLD phenotypes, it was noted that alanine (VIP > 1.5) may play a role as a distinguishing variable between groups. Additionally, they noted decreased glycine levels in individuals with MAFLD without fibrosis, but the ROC area was below 0.7, and the VIP scores were <2.0, so this measure did not distinguish between MAFLD phenotypes. In their work, they also found that increased levels of phenylalanine, tyrosine, alanine, and leucine occur in children with MAFLD and liver fibrosis [68].

### 4.3. Hypertension (HA) and Cardiovascular Diseases (CVDs) in Obesity—The Role of Metabolomics

An increase in body weight of only 10% is associated with an increase in the risk of developing HA by as much as 70%. The pathogenesis of HA in the course of obesity is multifaceted and includes, among others, excessive activity of the sympathetic nervous system, hyperleptinemia, increased sodium reabsorption in the renal tubules, increased intraabdominal pressure, stimulation of the RAA system, insulin resistance, or a generalized inflammatory process in the body. An increase in blood pressure can further lead to CVD and thus also endanger the lives of obese patients [75].

In 2016, Ruiz-Canela et al. published the results of their research on a large group of adults (970 people), during which one of the hypotheses was the assumption that baseline BCAA levels could be predictors of future CVD. During the study, they confirmed the hypothesis and noted that higher BCAA levels significantly correlated with CVD, especially stroke. At the same time, the study population had a large age range, which also allowed us to exclude the correlation of baseline BCAA levels with the patient’s age. Interestingly, only the correlation of isoleucine with CVD remained statistically significant after adjusting the analysis for the patients’ diabetic status, HA occurrence, or dyslipidemia [69].

In the same year, Hao et al. presented the results of their study, in which they assessed the effect of metabolomics on the development of hypertension in adult Chinese citizens. Their study was part of the China Multiprovincial Cohort Study. From 1133 participants of the population-based study who did not have hypertension at baseline, they selected 55 who developed hypertension during the 5-year follow-up. Then, 34 were randomly selected from this group and matched with 34 control subjects. After the initial assessment, 29 HA subjects and 29 control subjects were qualified for the study. During the study, 241 metabolites were measured, and correlations between their baseline levels and the risk of HA were sought. It turned out that 26 metabolites, including 8 amino acids, 7 carbohydrates, 4 carboxylic acids, 3 phenols, and 4 other metabolites, showed differences in their baseline levels between the control and study groups. After taking into account variables such as BMI, smoking, and alcohol consumption, only 16 metabolites showed statistical significance with the risk of developing HA. Among these metabolites was phenylalanine (OR: 0.49, 95% CI: 0.26–0.91, *p* = 1.12 × 10^−2^), which was negatively associated with the risk of hypertension. It also correlated with the metabolites tyrosine (r = 0.73) and noradrenaline (r = 0.39). Additionally, the researchers performed a pathway enrichment analysis using the 26 most significant metabolites. As a result, they identified three new metabolic pathways that were associated with HA risk: phenylalanine metabolism, tyrosine metabolism, and tryptophan biosynthesis [70].

In 2018, Tobias et al. published a study examining a large group of middle-aged women in the US. The study focused on finding potential CVD markers among BCAAs. They found that higher total BCAA levels were associated with a higher risk of CVD, which was consistent with the previous study by Ruzi-Canela et al. Analyzing the correlation of individual amino acids with CVD, leucine was found to be weaker than isoleucine and valine. In addition, multivariate models were also analyzed between BCAA quintiles and CVD. The results of the analysis showed that BCAAs as a whole, isoleucine, leucine, and valine increased the risk of CVD by 31%, 37%, 18%, and 29%, respectively. These correlations remained statistically significant except for leucine, for which the trend was *p* = 0.07. Additionally, it was noted that the association of BCAAs with CVD was significantly higher when CVD was concomitant with T2DM. The researchers also observed no difference in the correlation between BCAA and CVD with long-term follow-up, suggesting that baseline BCAA levels may be a predictive marker of CVD risk in short- and long-term follow-up [71].

Flores-Guerrero et al., in their 2019 study, also focused on the search for the correlation of BCAA levels with the occurrence of HA. This relationship was examined using univariate and multivariate linear regression analysis. In the multivariate analysis, a positive correlation of BCAA with, among others, diastolic blood pressure and BMI was noted. During over 8 years of observation, 924 people were diagnosed with HA de novo, which significantly correlated with increased BCAA levels in these people. Cox regression analysis confirmed the observed relationships both in the crude model and after adjustment for BMI, T2DM, cigarette smoking, alcohol consumption, positive family history of HA, and lipid profile. Additionally, the HA risk prediction model, including BCAA risk factors, was assessed—the C index was 0.809. After adding the total BCAA concentration to it, the C index slightly increased to 0.811, which indicates an improvement in its predictive ability in terms of NRI [72].

In the same year, the article by Chen et al. was published, who went a step further and decided to check the indirect effect of BCAAs, through their metabolites, on the development of HA. An example is β-Hydroxy β-methylbutyric acid (HMB), which is a metabolite of leucine and, as a precursor of cellular cholesterol synthesis, may have an impact on lipid disorders in obese people. In the study by Chen et al., the research group supplemented HMB for 3–8 weeks. After this time, a decrease in cholesterol and LDL levels was observed. In addition, it was noted that after supplementation, systolic blood pressure (SBP) in patients dropped by about 4 mmHg; such an observation gives hope for the cardioprotective function of HMB. In addition, it was noted that reduced urinary sodium excretion, which may contribute to HA, was associated with increased serum HMB concentration [73].

In 2022, Arjmand et al. published the results of a randomized clinical trial in a large group of patients with different stages of HA. The researchers assessed the relationship between the levels of acylcarnitines and amino acids and the occurrence of HA. During numerous analyses, they observed that patients with stage 2 HA had higher median levels of alanine, leucine, phenylalanine, valine, and proline than patients with normal RR (*p* ≤ 0.025). In contrast, the researchers found lower serum levels of glycine and serine in patients with stage 2 HA than in patients without HA (*p* ≤ 0.045). Additionally, in their work, Arjmand et al. drew attention to an index consisting of glycine and serine, which can predict the occurrence of stage 2 HA regardless of risk factors [74].

### 4.4. Summary

In the above-mentioned studies, significant correlations between individual metabolites and obesity complications were indicated. The list of metabolites with the number of works in which their relationships with MAFLD, T2DM, or HA were described is presented in Table 2.

## 5. Limitations

The above review should take into account certain limitations. For example, the number of original papers on metabolomics is limited when it comes to human studies. A much larger group of studies concerns animal models, which may not be as reliable as human serum testing. Some metabolites have not yet been properly studied in the human population, making it difficult to draw conclusions regarding their role as biomarkers. Moreover, the number of studies was significantly limited by the criterion of searching for studies on metabolomics but without therapeutic interventions in patients.

## 6. Conclusions and Future Management Strategies

Metabolomics holds the promise of finding the basic metabolic pathways and developing new predictive markers for obesity-related chronic diseases such as T2DM, MAFLD, HA, and CVD. Among studies on the correlation of metabolites with the aforementioned diseases, the greatest attention is focused on BCAAs, which seem to be the most sensitive markers of both obesity and chronic diseases. Depending on the disease, emphasis is placed on other BCAAs in combination with individual AAs. In T2DM, the most sensitive and specific seem to be the levels of leucine, isoleucine, valine, phenylalanine, and tyrosine, which increase with impaired glucose metabolism. Interestingly, several studies have noted that the above-mentioned metabolites do not affect pancreatic β-cells but insulin uptake by tissues, which is responsible for the basic mechanism occurring in insulin resistance. However, many researchers point out that the results should account for the gender, degree of sexual maturity, and ethnic origin of the subjects in order to be reliable because it seems that the concentration of these metabolites is influenced by both diet and the economy of human sex hormones.

In the case of MAFLD, it seems that predictive or diagnostic markers should be sought among BCAAs (especially valine) and alanine, phenylalanine, and tyrosine. Their high concentration may indicate a disease process, and the higher the level, the greater the advancement associated with the appearance of liver fibrosis. However, due to the limited analytical value of BCAAs considered in isolation, it is worth developing diagnostic models that would help predict the development of MAFLD or detect it at an early stage. Early diagnosis makes it possible to cure the changes before fibrosis occurs, i.e., irreversible changes.

Obesity is also inevitably associated with hypertension and cardiovascular diseases. Among the metabolites, it seems that isoleucine and leucine are mainly associated with an increased risk of developing these diseases. However, in the case of these complications of obesity, there is a deficit of research on children and adolescents who are increasingly diagnosed with obesity-related hypertension.

During the review of the literature, attention is also drawn to the gap in the form of research on the role of adipokines in chronic diseases. It is known that adipokines play a major role in the pathogenesis of obesity, but no precise correlations have yet been found between specific adipokines and T2DM, MAFLD, or CVD.

In order to introduce metabolites such as BCAAs or AAs into the common diagnostics of chronic diseases in the course of obesity, it would be necessary to first develop specific biochemical test panels that would combine BCAAs, AAs, and classic markers of individual diseases. Metabolomics panels containing BCAAs, adipokines, and classical markers (e.g., fasting glucose) could be used as diagnostic tools in everyday clinical practice. These panels may help to identify individuals at risk of complications such as T2DM and MAFLD earlier than conventional methods. However, more research is needed to determine which metabolites, to what extent, and with what advance, can be predictive markers of obesity complications. Research should be extended primarily to the group of children and adolescents who are developing eating habits and who are currently experiencing the greatest increase in obesity.

## Figures and Tables

**Table 1:** Summary of the role of metabolites and complications in the course of obesity based on the available literature.

Author, Year	Age [Years]	Cohort Size	Metabolites	Obesity Complications	Significant Differences and Correlations
Newbern D. et al., 2014 [54].	12–18	82	adiponectin, leptin, glutamine, leucine, isoleucine, valine	T2DM	A positive effect of BCAAs was noted with HOMA-IR (*p* = 0.0115). Differences in correlations between BCAAs and insulin resistance were noted between male and female subjects.
Lee A. et al., 2015 [55].	10–12	109	alanine, glycine, serine, leucine, isoleucine, valine, phenylalanine, tyrosine, lysine	T2DM	BCAAs have a significant positive correlation with HOMA-IR (r = 0.45, *p* < 0.001). Obese children have increased levels of BCAAs (*p* ≤ 0.0002), phenylalanine (*p* = 0.0164), and tyrosine (*p* = 0.0002). As obesity progresses, glycine (*p* < 0.0001) and serine (*p* = 0.0231) levels decrease.
Tillin T. et al., 2015 [56].	40–69	2286 (1279 Europeans and 1007 Asians)	alanine, glycine, isoleucine, phenylalanine, valine, tyrosine	T2DM	Positive correlations of isoleucine (r = 0.20, *p* < 0.001), leucine (r = 0.18, *p* < 0.001), valine (r = 0.17, *p* < 0.001), phenylalanine (r = 0.19, *p* < 0.001), tyrosine (r = 0.22, *p* < 0.001), and alanine (r = 0.15, *p* < 0.001) were observed with markers of glucose metabolism disorders. Glycine (r = −0.20, *p* < 0.001) and glutamine (r = −0.15, *p* < 0.001) remained negatively correlated with markers of glycemia and insulin resistance.
Chent. et al., 2016 [57].	20–75	429	leucine, isoleucine, valine, phenyloalanine	T2DM	Biomarkers of T2DM include leucine (HR = 2.09, 95% CI: 1.25–3.50), isoleucine (HR = 2.00, 95% CI: 1.20–3.33), valine (HR = 1.96, 95% CI: 1.18–3.25), phenylalanine (HR = 2.18, 95% CI: 1.30–3.65), and tyrosine (HR = 2.51, 95% CI: 1.50–4.20), which were higher at baseline in those diagnosed with T2DM after 10 years. The increase in AA levels outpaced the increase in conventional clinical markers of T2DM.
Honda M.S. et al., 2016 [58].	median age 52 years old	78	leucine, isoleucine, valine, tyrosine	T2DM	BCAAs have a positive effect on insulin sensitivity in nondiabetics (r = 0.30, *p* = 0.02), but their levels also correlate with increased fasting glucose (r = 0.25, *p* = 0.03) and insulin levels (r = 0.28, *p* = 0.02), which may modify their relationship with insulin resistance in obesity and T2DM. BCAA levels are also related to muscle mass (r = 0.35, *p* = 0.01). Tyrosine does not affect tissue insulin sensitivity (r = −0.05, *p* = 0.75).
Haufe S. et al., 2016 [59].	median age 44 years old	111	leucine, isoleucine, valine, tyrosine, phenylalanine	T2DM, MAFLD	BCAA levels are gender-dependent; in women, they are initially lower than in men (*p* < 0.01). A significant correlation was noted between tissue sensitivity to insulin and BCAA levels (r = −0.45, *p* < 0.001), as well as a statistically significant effect of isoleucine (r = 0.38, *p* = 0.002) and valine (r = 0.35, *p* = 0.004) on liver resistance to insulin. Additionally, it was noted that increased levels of the metabolites studied (except phenylalanine) significantly affect the increased accumulation of intrahepatic lipids (r = 0.40, *p* < 0.001).
Goffredo M. et al., 2017 [60].	10–16	78	lysine, leucine, isoleucine, valine	T2DM, MAFLD	It has been noted that isoleucine (r = −0.32, *p* < 0.01) and valine (r = −0.29, *p* < 0.01) show a negative correlation with tissue insulin sensitivity. Additionally, high BCAA levels are associated with worse hepatic glucose metabolism and hepatic insulin sensitivity (r = −0.35, *p* < 0.01). Obese individuals with MAFLD have higher levels of valine, leucine, and isoleucine. The greatest attention is paid to the correlation of valine with MAFLD, the level of which was significantly higher in those with increased fatty liver disease during the 3-year follow-up (*p* < 0.001).
Flores-Guerrero JL et al., 2018 [61].	28–75	6244	leucine, isoleucine, valine	T2DM	A positive effect of BCAAs (valine (HR = 1.45, 95% CI: 1.25–1.68, *p* < 0.001), isoleucine (HR = 1.47, 95% CI: 1.27–1.70, *p* < 0.001), and leucine (HR = 1.39, 95% CI: 1.20–1.61, *p* < 0.001)) on the increased risk of insulin resistance and T2DM has been noted. At the same time, no association has been found between these BCAAs and the function of pancreatic β-cells (*p* > 0.05).
Perng W. et al., 2022 [62].	8–14	179	leptin, alanine, leucine, isoleucine, valine, phenylalanine, tyrosine	T2DM	Strong correlations were noted between isoleucine (r = 0.30, *p* = 0.01), tyrosine (r = 0.28, *p* = 0.02), and phenylalanine (r = 0.25, *p* = 0.03) and insulin resistance. Additionally, differences in metabolic profiles were observed depending on gender (*p* < 0.01) and puberty (*p* < 0.05).
Jin, R. et al., 2016 [63].	11–17	39	alanine, glycine, serine, leucine, isoleucine, valine, tyrosine	MAFLD	A strong positive association with MAFLD was demonstrated, as well as less significant correlations for BCAAs, glycine, serine, and alanine metabolism in obese individuals with MAFLD (*p* < 0.05).
Gaggini, M. et al., 2018 [64].	18–65	64	alanine, isoleucine, valine, serine, glycine, glutathione	MAFLD	Increased levels of valine, isoleucine, and alanine have been observed in patients with MAFLD (*p* < 0.001). Changes in glutathione, serine, and glycine levels have also been reported in obese MAFLD patients (*p* < 0.05), and a new GSG index has been proposed that could reflect the stage of MAFLD (r = 0.70).
Romero-Ibarguengoitiam. et al., 2018 [65].	18–45	137	alanine, glycine, leucine, phenylalanine, tyrosine, valine	MAFLD	Higher concentrations of alanine (*p* = 0.006), leucine (*p* = 0.022), phenylalanine (*p* = 0.005), tyrosine (*p* < 0.001), and valine (*p* = 0.004) were noted in obese individuals with MAFLD than in obese individuals without MAFLD.
Lischka J. et al., 2021 [66].	9–19	68	alanine, glycine, tyrosine, phenyloalanine, BCAA	MAFLD	BCAAs have been shown to be significantly correlated with MAFLD (r = 0.46, *p* < 0.01), and a linear regression model was developed that could predict the risk of developing MAFLD in patients.
Chae W. et al., 2022 [67].	6–19	165	leucine, isoleucine, valine, lysine, tyrosine	MAFLD	BCAA (*p* = 0.03), lysine, and tyrosine (*p* = 0.039) levels were shown to be significantly higher in obese patients with MAFLD than in obese patients without MAFLD. Additionally, important metabolites were indicated in predicting the development of MAFLD: valine (*p* = 0.005), tyrosine (*p* = 0.003), glutamic acid (*p* = 0.006), and glycine.
Garibay-Nieto N. et al., 2023 [68].	8–16	79	alanine, glycine, leucine, valine, phenylalanine, tyrosine	MAFLD	Glycine (*p* < 0.05) and alanine (*p* < 0.05) may be the distinguishing variables between MAFLD patients with and without liver fibrosis. Additionally, higher levels of phenylalanine (*p* < 0.01), tyrosine (*p* < 0.01), and leucine (*p* < 0.05) may be observed in MAFLD + fibrosis patients.
Ruiz-Canela M. et al., 2016 [69].	median age 67 years old	970	leucine, isoleucine, valine	CVD	An association of CVD with higher baseline BCAA concentrations was confirmed in obese individuals (95% CI: 1.18–1.29, *p* < 0.0001).
Hao Y. et al., 2016 [70].	median age 52 years old	58	treonine, phenyloalanine, glycine, tyrosine	HA	Phenylalanine and tyrosine metabolism may be negatively correlated with the risk of HA in adults (95% CI = 0.16–0.74, *p* = 0.006).
Tobias D.K., 2018 [71].	median age 54 years old	27,041	leucine, isoleucine, valine	HA	Higher levels of BCAAs correlate with higher risk of CVD (95% CI = 1.08–1.18, *p* < 0.0001). Additionally, the correlation of leucine (95% CI: 1.07 to 1.17, *p* < 0.0001) with CVD appears to be weaker than the correlation of isoleucine (95% CI: 1.09 to 1.19, *p* < 0.0001) and valine (95% CI: 1.08 to 1.18, *p* < 0.0001) with CVD. The association of BCAAs with CVD was stronger in the presence of T2DM.
Flores-Guerrero, J.L. et al., 2019 [72].	median age 49 years old	4169	leucine, isoleucine, valine	HA	High BCAA concentrations have been reported to correlate with an increased risk of hypertension in middle-aged people, regardless of gender (95% CI = 1.07–1.30, *p* = 0.001).
Chen L. et al., 2019 [73].	30–75	64	leucine, isoleucine, valine	HA	It has been suggested that BCAAs may also indirectly induce HA through their metabolites, e.g., HMB, with the increase in the concentration in the body positively correlating with a reduction in SBP (*p* < 0.001).
Arjmand B. et al., 2023 [74].	52–60	1200	alanine, leucine, phenylalanine, tyrosine, valine, serine, lysine, glycine	HA	Alanine, leucine, phenylalanine, valine, and proline levels were higher in subjects with HA grade 2 (*p* < 0.05). Glycine and serine levels were lower in patients with HA than in those without HA (*p* < 0.05).

Abbreviations: T2DM, type 2 diabetes; MAFLD, metabolic fatty liver disease; BCAAs, branched-chain amino acids; AAs, amino acids; HOMA-IR, Homeostasis Model Assessment of Insulin Resistance; CVD, cardiovascular disease; HA, hypertension; SBP, systolic blood pressure; GSG index, (glutamate/[serine + glycine]); HMB, β-hydroxyisovalerate β-hydroxy-β-methylbutyric acid.

**Table 2:** Metabolites and their possible association with MAFLD, T2DM, or HA.

Metabolites	Number of Studies Included to Review	Cohort Details	Results
Glycine	8	children, adolescents, adults	Lower levels are correlated with HA and MAFLD.
Alanine	8	children, adolescents, adults	Higher levels are correlated with MAFLD and HA stage 2.
Glutamine	2	adolescents, adults	Negative correlation with insulin resistance.
Valine	19	children, adolescents, adults	High levels are correlated with MAFLD and IR.
Serine	3	children, adults	Lower levels are correlated with insulin resistance.
Lysine	4	children, adolescents, adults	Higher levels are in patients with MAFLD.
Tyrosine	12	children, adolescents, adults	Strong correlation with MAFLD, T2DM, and hepatic metabolism disorders.
Phenylalanine	10	children, adolescents, adults	Higher levels are correlated with MAFLD and T2DM.
Leucine	16	children, adolescents, adults	Higher levels are correlated with a risk of MAFLD and insulin resistance.
Isoleucine	16	adolescents, adults	Strong correlation with insulin resistance and risk of T2DM.
Leptin	2	adolescents	Strong correlation with obesity.
Adiponectin	1	adolescents	Negative correlation with HOMA-IR. A low level is probably a marker of metabolic disorders.

Abbreviations: HA, arterial hypertension; MAFLD, metabolic fatty liver disease; IR, insulin resistance; T2DM, diabetes mellitus type 2; HOMA-IR, Homeostasis Model Assessment of Insulin Resistance.
